# Supplementary material for: Modeling glioblastoma heterogeneity as a dynamic network of cell states
Source: Mol Syst Biol. 2021 Sep 16;17(9):e10105. doi: 10.15252/msb.202010105 (PMC8444284; doi:10.15252/msb.202010105)
Supplement: Supplementary file 6 — Source Data for Figure 5 [file MSB-17-e10105-s004.zip › Figure5A_sourcedata/GSEA_3071/nosigdb_stateIV.GseaPreranked.1622795164626/SUVA_NPC1_PMID_31327527.html]

Details for gene set SUVA\_NPC1\_PMID\_31327527[GSEA]

|  || Dataset | state53071 |
| Phenotype | NoPhenotypeAvailable |
| Upregulated in class | na\_pos |
| GeneSet | SUVA\_NPC1\_PMID\_31327527 |
| Enrichment Score (ES) | 0.7701629 |
| Normalized Enrichment Score (NES) | 3.442968 |
| Nominal p-value | 0.0 |
| FDR q-value | 0.0 |
| FWER p-Value | 0.0 |
Table: GSEA Results Summary

  

Fig 1: Enrichment plot: SUVA\_NPC1\_PMID\_31327527      
 Profile of the Running ES Score & Positions of GeneSet Members on the Rank Ordered List

  

| PROBE | GENE SYMBOL | GENE\_TITLE | RANK IN GENE LIST | RANK METRIC SCORE | RUNNING ES | CORE ENRICHMENT || 1 | DCX |  |  | 4 | 2.752 | 0.0717 | Yes |
| 2 | BEX1 |  |  | 18 | 2.252 | 0.1286 | Yes |
| 3 | FXYD6 |  |  | 19 | 2.252 | 0.1879 | Yes |
| 4 | ASCL1 |  |  | 53 | 1.813 | 0.2294 | Yes |
| 5 | MAP2 |  |  | 55 | 1.811 | 0.2770 | Yes |
| 6 | SOX11 |  |  | 56 | 1.811 | 0.3247 | Yes |
| 7 | TAGLN3 |  |  | 71 | 1.720 | 0.3673 | Yes |
| 8 | DLL3 |  |  | 105 | 1.521 | 0.4011 | Yes |
| 9 | LRRN1 |  |  | 122 | 1.482 | 0.4371 | Yes |
| 10 | CHD7 |  |  | 144 | 1.386 | 0.4696 | Yes |
| 11 | TUBA1A |  |  | 165 | 1.337 | 0.5011 | Yes |
| 12 | TUBB3 |  |  | 167 | 1.330 | 0.5359 | Yes |
| 13 | TCF12 |  |  | 176 | 1.303 | 0.5687 | Yes |
| 14 | BTG2 |  |  | 200 | 1.260 | 0.5976 | Yes |
| 15 | HES6 |  |  | 250 | 1.150 | 0.6185 | Yes |
| 16 | PFN2 |  |  | 270 | 1.107 | 0.6441 | Yes |
| 17 | SOX4 |  |  | 377 | 0.928 | 0.6484 | Yes |
| 18 | OLIG1 |  |  | 391 | 0.914 | 0.6700 | Yes |
| 19 | MARCKSL1 |  |  | 417 | 0.888 | 0.6886 | Yes |
| 20 | ETV1 |  |  | 488 | 0.824 | 0.6970 | Yes |
| 21 | ABAT |  |  | 515 | 0.805 | 0.7132 | Yes |
| 22 | CD24 |  |  | 545 | 0.770 | 0.7280 | Yes |
| 23 | HIP1 |  |  | 563 | 0.753 | 0.7446 | Yes |
| 24 | GLCCI1 |  |  | 611 | 0.719 | 0.7546 | Yes |
| 25 | SCG3 |  |  | 632 | 0.707 | 0.7694 | Yes |
| 26 | PCBP4 |  |  | 719 | 0.649 | 0.7702 | Yes |
| 27 | PTPRS |  |  | 1253 | 0.451 | 0.6806 | No |
| 28 | SERINC5 |  |  | 1383 | 0.421 | 0.6671 | No |
| 29 | DLL1 |  |  | 1463 | 0.399 | 0.6626 | No |
| 30 | LMF1 |  |  | 1749 | 0.339 | 0.6173 | No |
| 31 | DBN1 |  |  | 1846 | 0.325 | 0.6076 | No |
| 32 | MLLT11 |  |  | 1932 | 0.308 | 0.5995 | No |
| 33 | NXPH1 |  |  | 1950 | 0.306 | 0.6043 | No |
| 34 | ELMO1 |  |  | 1966 | 0.303 | 0.6095 | No |
| 35 | SEZ6L |  |  | 2065 | 0.287 | 0.5984 | No |
| 36 | MYT1 |  |  | 2292 | 0.257 | 0.5621 | No |
| 37 | GRIK2 |  |  | 2858 | -0.314 | 0.4629 | No |
Table: GSEA details [plain text format]

  

Fig 2: SUVA\_NPC1\_PMID\_31327527: Random ES distribution      
 Gene set null distribution of ES for **SUVA\_NPC1\_PMID\_31327527**
